# The AGE-RAGE axis associates with chronic pulmonary diseases and smoking in the Rotterdam study

**DOI:** 10.1186/s12931-024-02698-1

**Published:** 2024-02-09

**Authors:** Tianqi Lu, Lies Lahousse, Sara Wijnant, Jinluan Chen, Guy G. Brusselle, Mandy van Hoek, M. Carola Zillikens

**Affiliations:** 1 Department of Internal Medicine, Erasmus University Medical Center, ‘s-Gravendijkwal 230, 3015GD, Rotterdam, The Netherlands; 2 Department of Respiratory Medicine, Ghent University Hospital, Ghent, Belgium; 3 Department of Epidemiology, Erasmus University Medical Center, Rotterdam, The Netherlands; 4 Department of Bioanalysis, Faculty of Pharmaceutical Sciences, Ghent University, Ghent, Belgium; 5 Department of Respiratory Medicine, Erasmus University Medical Center, Rotterdam, The Netherlands

**Keywords:** Advanced glycation end products (AGEs), Skin autofluorescence (SAF), Chronic obstructive pulmonary disease (COPD), Spirometry, Lung function

## Abstract

**Background:**

Chronic obstructive pulmonary disease (COPD) and asthma associate with high morbidity and mortality. High levels of advanced glycation end products (AGEs) were found in tissue and plasma of COPD patients but their role in COPD and asthma is unclear.

**Methods:**

In the Rotterdam Study (*n* = 2577), AGEs (by skin autofluorescence (SAF)), FEV_1_ and lung diffusing capacity (D_LCO_c and D_LCO_c /alveolar volume [V_A_]) were measured. Associations of SAF with asthma, COPD, GOLD stage, and lung function were analyzed using logistic and linear regression adjusted for covariates, followed by interaction and stratification analyses. sRAGE and EN-RAGE associations with COPD prevalence were analyzed by logistic regression.

**Results:**

SAF associated with COPD prevalence (OR = 1.299 [1.060, 1.591]) but not when adjusted for smoking (OR = 1.106 [0.89, 1.363]). SAF associated with FEV_1_% predicted (β=-3.384 [-4.877, -1.892]), D_LCO_c (β=-0.212 [-0.327, -0.097]) and GOLD stage (OR = 4.073, *p* = 0.001, stage 3&4 versus 1). Stratified, the association between SAF and FEV_1_%predicted was stronger in COPD (β=-6.362 [-9.055, -3.670]) than non-COPD (β=-1.712 [-3.306, -0.118]). Association of SAF with D_LCO_c and D_LCO_c/V_A_ were confined to COPD (β=-0.550 [-0.909, -0.191]; β=-0.065 [-0.117, -0.014] respectively). SAF interacted with former smoking and COPD prevalence for associations with lung function. Lower sRAGE and higher EN-RAGE associated with COPD prevalence (OR = 0.575[0.354, 0.931]; OR = 1.778[1.142, 2.768], respectively).

**Conclusions:**

Associations between SAF, lung function and COPD prevalence were strongly influenced by smoking. SAF associated with COPD severity and its association with lung function was more prominent within COPD. These results fuel further research into interrelations and causality between SAF, smoking and COPD.

**Take-home message:**

Skin AGEs associated with prevalence and severity of COPD and lung function in the general population with a stronger effect in COPD, calling for further research into interrelations and causality between SAF, smoking and COPD.

**Supplementary Information:**

The online version contains supplementary material available at 10.1186/s12931-024-02698-1.

## Introduction

Chronic obstructive pulmonary disease (COPD) and asthma are chronic airway diseases causing substantial morbidity and mortality. COPD is characterized by respiratory symptoms and airflow limitation due to airway and/or alveolar abnormalities, caused by exposure to noxious particles or gases [1], most commonly cigarette smoke [2, 3] and is accompanied by systemic inflammation and oxidative stress [4]. Asthma is characterized by chronic inflammation induced airflow obstruction and has a strong genetic disposition [5, 6].

Advanced glycation end products (AGEs) are a heterogeneous group of molecules produced from non-enzymatic attachment of sugars to proteins, lipids and nuclear acids in the classical Maillard reaction [7, 8] They are linked to presence and induction of inflammation and cellular dysfunction [9]. AGEs contribute to aging and age-related diseases [10–12] by forming cross-links between proteins [13], modifying protein structure and functions [14], and via their receptor (RAGE) to induce inflammation [9, 15]. AGE accumulation accelerates with hyperglycemia, oxidative stress, chronic inflammation [13, 15], chronic kidney disease, exposure to high AGE containing foods [16] and tobacco smoke [17]. The AGE-RAGE axis may contribute to COPD development or may increase due to COPD associated inflammation. Smoking, a major risk factor for both COPD and AGE formation, may affect the association between AGEs and lung function.

Skin AGE measurement by autofluorescence (SAF) has recently been used as a marker for long-term AGE accumulation because of the long half-life (15 years) [18, 19] of skin collagen, to which AGEs bind. SAF was higher in COPD [12, 20, 21] compared to healthy controls although no associations were observed between AGEs in plasma and sputum and COPD or lung function values [21]. Genome-wide association identified a genetic variant in the RAGE ligand-binding domain (G82S) that correlated with forced expiratory volume in one second (FEV_1_) [22, 23]. RAGE-blocking was proposed as protective in COPD [24, 25]. Soluble forms of RAGE, collectively known as sRAGE [26], were significantly lower in COPD in previous studies [27, 28]. It was also found that sRAGE was a protective factor for the presence and severity of emphysema among CC-genotyped COPD patients of rs2070600 on the RAGE gene [29] and that lower sRAGE is associated with more severe airflow obstruction, heterogeneous distribution of emphysema, centrilobular emphysema, and 5-year progression of emphysema [30]. RAGE may also play a role in asthma, via IL-33 release and ILC2 accumulation which promote allergic airway disease [31]. sRAGE and another extracellular RAGE binding protein EN-RAGE [32] have also been described in asthma and lung inflammation [33–35]. However, no data on the association between SAF and asthma are available.

Taken together, AGEs and their interaction with RAGE might be involved in COPD and asthma, but large scale, in-depth population data are needed, including the role of smoking. We investigated the association of SAF, sRAGE, and EN-RAGE with COPD on the one hand and the association of SAF and asthma on the other hand as primary outcomes, and lung function parameters as secondary outcomes in the Rotterdam Study and studied the role of smoking in these associations.

## Methods

### Standard protocol approvals, registrations, and patient consents

The Rotterdam Study (RS) has been approved by Erasmus MC Medical Ethics Committee (registration number MEC 02.1015), executed by the Dutch Ministry of Health, Welfare and Sports (Population Screening Act WBO, license number 1071272-159521-PG). All participants gave written informed consent.

### Study population

Participants in our analyses originated from the RS, a population-based cohort study, initiated in 1990. Inhabitants of the Rotterdam suburb Ommoord aged ≥ 55 years were invited to participate. The first subcohort (RS-I) started in 1990, including 7983 participants of 55 years and over. In 2000, a second subcohort (RS-II) started with 3011 participants aged 55 years and over. The third subcohort (RS-III) including another 3932 participants of 45 years and over started in 2006. The fourth subcohort (RS-IV) was established in 2016. All participants were examined at baseline and at follow up every 3–5 years. The RS has been extensively described [36].

### Measurement of SAF

The AGE Reader™ (DiagnOptics B.V., Groningen, The Netherlands) measures AGE content of the skin at the inner part of dominant forearm. The measurement is based on fluorescent properties of AGEs [37] at a UV reflectance percentage (R%) higher than 6%. Participants with reflectance of 6% or lower are excluded. Details are described elsewhere [38]. The AGE Reader™ measured SAF in *n* = 3009 participants (754 in RS-I 6th follow-up, 1088 in RS-II 4th follow-up and 1167 in RS-III 2nd follow-up). SAF outliers exceeding mean ± 4SD (*N* = 8) were excluded, as were participants with missing lung function and D_LCO_c. Inclusion and exclusion are shown in supplementary Fig. 1.

### Measurements of sRAGE and EN-RAGE

sRAGE and EN-RAGE were measured in plasma collected between 1997 and 1999 from a random subset of 1208 participants of RS-I. Details of measurement were described previously [39]. Inclusion and exclusion are shown in supplementary Fig. 2.

### Spirometry measurements and COPD diagnosis

Lung functions and spirometry were conducted according to American Thoracic Society (ATS)**/**European Respiratory Society (ERS) guidelines [40, 41]. FEV_1_% predicted is the percentage of predicted FEV_1_, the expected value for the same sex, age, height and ethnicity estimated by the Global Lung Function Initiative (GLI) reference equations [41]. Spirometry D_LCO_ (mmol·min^− 1^·kPa^− 1^) and alveolar volume (V_A_) were measured using the single-breath technique. D_LCO_c is diffusing capacity of the lung measured by carbon monoxide corrected for hemoglobin levels (anemia). D_LCO_c/V_A_ is D_LCO_c divided by alveolar volume(V_A_) representing transfer efficiency. COPD was defined (FEV_1_/forced vital capacity (FVC) < 0.7). The Global initiative for Obstructive Lung Disease (GOLD) stages (1–4) indicate COPD severity [1] based on FEV_1_% predicted. GOLD 1: FEV_1_% predicted > = 80%, GOLD 2: 50% =< FEV_1_% predicted < 80%, GOLD 3: 30% =< FEV_1_% predicted < 50%, GOLD 4: FEV_1_% predicted < 30% [42].

### Asthma definition

Asthma was defined by physician’s diagnosis in the medical file as described [6].

### Assessment of covariates

Age was from time of SAF measurement. Smoking status was categorized as never, former, and current based on habits of cigarette, cigar, and pipe smoking assessed at RS-I 6th visit, RS-II 4th visit, and RS-III 2nd visit. Pack-years were computed as years times daily cigarettes, cigar, and pipe divided by 20. Physical activity using the LASA Physical Activity Questionnaire was expressed in metabolic equivalents hours per week [37]. BMI (in kg/m^2^) and estimated glomerular filtration rate (eGFR) were previously described [43]. Type 2 diabetes mellitus (T2DM) was defined as previously described [44]. Oral and inhaled corticosteroid use was from prescription data through automated pharmacy records.

### Statistical analyses

Statistical analyses were performed in SPSS (version 29.0). Normality was determined using histograms and Q–Q plots and data presented as mean (± SD) or median (interquartile range) respectively. Means of continuous variables between groups were compared via independent samples T-test and Mann–Whitney U-test for normal and non-normal variables, respectively. The X^2^ test served to compare means of categorical variables. SAF was entered as continuous variable in all analyses.

The associations of SAF with COPD or asthma prevalence were analyzed using binary logistic regression with COPD or asthma as the outcomes. Models were adjusted for confounders and risk factors of high SAF with model 1 adjusted for age, sex and RS subcohorts; model 2 additionally adjusted for T2DM, physical activity, eGFR, BMI, oral and inhaled corticosteroids; model 3 additionally adjusted for smoking status. The association between SAF and GOLD stage 1–4 was analyzed via multinomial logistic regression (GOLD stage 1 as reference), using the same models.

Associations of SAF with lung function were analyzed in multiple linear regression with lung functions as outcomes in models adjusted as above. Heteroscedasticity was determined by plotting linear regression residuals and predicted outcome values.

Two-way interactions of SAF with smoking and COPD were checked by adding interaction terms into linear regression model 3. Interaction P-values less than 0.05 were considered statistically significant.

sRAGE and EN-RAGE were analyzed continuously, per unit increase of log transformed values because of skewed distributions. Outliers, outside of the mean  ± 4SD range, were excluded. Cox proportional hazards and logistic regression were used for analyses of the association of sRAGE and EN-RAGE with COPD incidence and prevalence. Follow-up started at sampling and ended at COPD diagnosis, death, or end of the study period (June 1, 2017), whichever came first.

Stratified analyses evaluated disproportionate effects in predefined strata for COPD, smoking status, and pack-years of smoking. Sensitivity analyses were carried out in subjects not diagnosed with COPD and those diagnosed with asthma excluding COPD.

Missing values were imputed using multiple imputation. Predictive mean matching (PMM) was used [43], with 5 iterations. Sensitivity analysis was conducted to evaluate if the association between SAF with COPD and lung function parameters remained consistent after imputation.

### Results

#### Study population

In total, 2577 subjects (age 72.3 ± 9.3 y (mean ± SD), 55.5% female) with data on SAF, asthma or COPD, and lung function (FEV_1_% predicted) were included. 613 subjects were diagnosed with COPD and 215 with asthma at time of SAF measurement. In the total population, SAF was higher in subjects with COPD (2.50 ± 0.52 A.U., *p* < 0.001) than in those without (2.36 ± 0.47 A.U.). SAF was also higher in current (2.53 ± 0.57 A.U., *p* < 0.001) and former smokers(2.42 ± 0.48 A.U., *p* < 0.001) than in never smokers(2.30 ± 0.46 A.U., *p* < 0.001). COPD participants were older (75.3 ± 8.7 y vs. 71.4 ± 9.3 y), more often male (57.3% vs. 40.6%) and had lower FEV_1_% predicted (83.91 ± 18.3), D_LCO_c (7.64 ± 2.0) and D_LCO_c/V_A_ (1.36 ± 0.3) compared to non-COPD participants (FEV_1_% predicted (102.25 ± 16.1), D_LCO_c (7.85 ± 1.7) and D_LCO_c/V_A_ (1.53 ± 0.2)). Detailed descriptives are shown in Table 1 and Supplementary Tables 1–3.

**Table 1:** Demographic and clinical characteristics in total population and stratified by COPD diagnosis

Parameters	Total population	COPD	No COPD
N	2577	613 (23.8%)	1964 (76.2%)
Age(years)*	72.28 ± 9.29	75.29 ± 8.72	71.35 ± 9.26
Sex			
Male/n(%)*	1148 (44.5%)	351 (57.3%)	797 (40.6%)
Female/n(%)*	1429 (55.5%)	262 (42.7%)	1167 (59.4%)
RS cohort			
RS I	615 (23.9%)	213 (34.7%)	402 (20.5%)
RS II	923 (35.8%)	246 (40.1%)	677 (34.5%)
RS III	1039 (40.3%)	154 (25.1%)	885 (45.1%)
SAF*	2.39 ± 0.49	2.50 ± 0.52	2.36 ± 0.47
FEV_1_%predicted *	97.89 ± 18.36	83.91 ± 18.25	102.25 ± 16.08
D_LCO_c #	7.82 ± 1.71	7.64 ± 1.97	7.85 ± 1.68
D_LCO_c/V_A_ *	1.51 ± 0.23	1.36 ± 0.25	1.53 ± 0.22
N for sRAGE & EN-RAGE	1192	83(7.0%)	1109(93.0%)
sRAGE^+^	2.97 ± 1.63	2.54 ± 1.23	3.00 ± 1.66
EN-RAGE*	13.00 ± 8.22	15.89 ± 11.59	12.79 ± 7.87
Asthma	215 (8.3%)	34 (5.5%)	181 (9.2%)
Smoking Status (*N* = 2575)			
Never Smokers*	848(32.9%)	106 (17.3%)	742 (37.8%)
Ex-Smokers*	1483 (57.5%)	409 (66.7%)	1074 (54.7%)
Current Smokers*	244 (9.5%)	98 (16.0%)	146 (7.4%)
T2DM	389 (15.1%)	101 (16.5%)	288 (14.7%)
Oral corticosteroids^+^	139	43 (7.2%)	96 (5.0%)
Inhaled corticosteroids*	196	83 (13.8%)	113 (5.9%)

* COPD vs. No COPD p-value < 0.001 obtained from T-test and Chi-square

^+^ COPD vs. No COPD p-value < 0.05

^#^ COPD vs. No COPD p-value = 0.065

### Association between SAF and COPD/asthma in the total population (table 2)

**Table 2:** Logistic regression of the association between SAF and COPD (613/2577), SAF and asthma (181/1964) in total population

COPD(n/*N* = 613/2577)	OR (95% CI)	***P***-value
Model 1	1.281 (1.051 -1.562)	.014
Model 2	1.299 (1.060 -1.591)	.012
Model 3	1.106 (.897 - 1.363)	.348
**Asthma(n/N = 181/1964)**		
Model 1	1.111 (.791 - 1.560)	.544
Model 2	1.060(.705 - 1.592)	.780
Model 3	1.040 (.688 - 1.571)	.854

Model 1: Age, sex, Rotterdam Study subcohort adjusted

Model 2: Model 1 + diabetes, physical activity, eGFR, BMI, oral corticosteroids and inhaled corticosteroids prescription adjusted

Model 3: Model 2 + smoking status adjusted

Higher SAF was significantly associated with higher prevalence of COPD in model 2 (OR = 1.299, 95% confidence interval (CI) [1.060, 1.591]) but not after adjusting for smoking (model 3 OR = 1.106, CI [0.897, 1.363]). No significant association between SAF and asthma prevalence was found (OR = 1.097, CI [0.688, 1.571]).

### Association between SAF and lung function in the total population

Results from linear regression models are shown in Table 3. SAF inversely associated with FEV_1_% predicted (β= -3.384[95% CI -4.877, -1.892], *p* < 0.001) and D_LCO_c (β= -0.212[-0.327, -0.097], *p* < 0.001) in Model 3). Significant interactions for associations between SAF and FEV_1_% predicted, D_LCO_c and D_LCO_c/V_A_ were observed between SAF and smoking (*p* = 0.004; *p* <0.001; *p* = 0.03, respectively) and between SAF and COPD (*p* =0.006; *p* < 0.001; *p* < 0.001, respectively).

**Table 3:** Linear regression of the association between SAF and lung function parameters in total population

FEV1 predicted% (*N* = 2577)	Unstandardized coefficient β (95% CI)	***P***-value	***P*** for Interaction SAF*smoking^1^		P for Interaction SAF*COPD^2^
Model 1	-5.612 (-7.145 - -4.080)	< 0.001		Ex 0.004	0.006
Model 2	-4.544 (-6.037 - -3.052)	< 0.001			
Model 3	-3.384 (-4.877 - -1.892)	< 0.001			
**DLCOc(** *N* ** = 2437)**					
Model 1	− 0.326 (-0.439 - − 0.213)	< 0.001		Ex < 0.001	< 0.001
Model 2	− 0.299 (-0.414 - − 0.185)	< 0.001			
Model 3	− 0.212 (-0.327 - − 0.097)	< 0.001			
**DLCOc/VA(** *N* ** = 2437)**					
Model 1	− 0.027 (-0.046 - − 0.007)	0.007		Ex 0.03	< 0.001
Model 2	− 0.031 (-0.050 − 0.012)	0.002			
Model 3	− 0.015 (-0.034 − 0.004)	0.116			

Model 1: Age, sex, Rotterdam Study subcohort adjusted

Model 2: Model 1 + diabetes, physical activity, eGFR, BMI, oral and inhaled corticosteroids prescription adjusted

Model 3: Model 2 + smoking status adjusted

^1^*P* values for interaction were derived from the model with interaction terms of SAF and smoking status

^2^*P* values for interaction were derived from the model with interaction terms of SAF and COPD diagnosis

### Association between SAF and COPD GOLD Stage

Results from multinomial logistic regression for 613 COPD subjects are shown in Table 4. Higher SAF associated with more severe COPD GOLD stage compared to stage 1 after adjusting for all potential confounders (GOLD stage 2 (OR = 2.325[1.577, 3.429]), GOLD stage 3&4 (OR = 4.073[1.752, 9.468])) (Model 3, Table 4).

**Table 4:** Multinomial logistic regression of the association between SAF and GOLD Stage 1–4(N = 613)

**Model 1**	**OR (95% CI)**	***P-*** **value**
Stage 2 (*N* = 197)	2.644 (1.841–3.798)	< 0.001
Stage 3&4 (*N* = 26)	4.777 (2.343–10.175)	< 0.001
**Model 2**		
Stage 2 (*N* = 197)	2.507 (1.719–3.655)	< 0.001
Stage 3&4 (*N* = 26)	4.694 (2.053–10.736)	< 0.001
**Model 3**		
Stage 2 (*N* = 197)	2.325 (1.577–3.429)	<0.001
Stage 3&4 (*N* = 26)	4.073 (1.752–9.468)	0.001

Multinomial Logistic Regression (Stage 1 (*N* = 390) as reference category)

Model 1: Age, sex, Rotterdam Study subcohort adjusted

Model 2: Model 1 + diabetes, physical activity, eGFR, BMI, oral and inhaled corticosteroids prescription adjusted

Model 3: Model 2 + smoking status adjusted

### Associations between sRAGE, EN-RAGE and COPD prevalence and incidence 

Serum sRAGE (ng/mL) was significantly lower (2.54 ± 1.23 vs. 3.00 ± 1.66) and EN-RAGE (ng/mL) was significantly higher in COPD than in non-COPD participants (15.89 ± 11.59 vs. 12.79 ± 7.87). Cox proportional hazards and logistic regression analyses are shown in Table 5. Significant associations were found for sRAGE and EN-RAGE and COPD prevalence (OR = 0.575[0.354, 0.931], *p* = 0.025; OR = 1.778[1.142, 2.768], *p* = 0.011 respectively). 151 participants developed COPD during a median 10.9 years of follow up. There was no significant association between serum sRAGE nor EN-RAGE and COPD incidence.

**Table 5:** Cox proportional analyses for sRAGE, EN-RAGE and COPD incidence and prevalence

	COPD incidence sRAGE n/*N* = 151/1114, EN-RAGE n/*N* = 151/1110		COPD prevalence sRAGE and EN-RAGE n/*N* = 83/1192	
**sRAGE**	HR (95% CI)	*P*-value	OR (95% CI)	*P*-value
Model 1	1.051 (0.745 -1.484)	0.776	0.597(0.377 - 0.947)	0.028
Model 2	1.028 (0.720 -1.467)	0.878	0.575(0.354 - 0.931)	0.025
**EN-RAGE**	HR (95%CI)	*P*-value	OR (95% CI)	*P*-value
Model 1	1.066 (0.766 - 1.482)	0.705	1.735(1.124 - 2.678)	0.013
Model 2	1.037 (0.739 - 1.455)	0.834	1.778(1.142 - 2.768)	0.011

*sRAGE and EN-RAGE was used as Ln-transformed value in the models

Model 1: Age, sex adjusted

Model 2: Model 1 + diabetes, eGFR, BMI and smoking status adjusted

### Stratified analyses

Stratified analyses by COPD status for associations between SAF and lung function are shown in Table 6. FEV_1_% predicted was available in *n* = 2577 including 613 COPD participants; D_LCO_c data and D_LCO_c/V_A_ were available in *n* = 2356 including 323 COPD participants. Effect sizes for the inverse association between SAF and FEV_1_% predicted were larger in COPD (β=-6.362[95% CI -9.055, -3.670], *p* < 0.001) than in non-COPD participants (β=-1.712[-3.306, -0.118], *p* = 0.035) in Model 3. There was a significant association between SAF and D_LCO_c, D_LCO_c/V_A_ in COPD participants in Model 3 (β =-0.550[-0.909, -0.191], *p* = 0.003; β =-0.065[-0.117, -0.014], *p* = 0.013 respectively), which was not significant in non-COPD participants (β =-0.112[-0.232, 0.009], β = 0.001[-0.020, 0.021] respectively).

**Table 6:** Linear regression of the association between SAF and lung function parameters stratified by COPD diagnosis

FEV1%predicted *N* = 2577	No COPD (*N* = 1964)		COPD (*N* = 613)	
	*Unstandardized coefficient β (95% CI)*	*P-value*	*Unstandardized coefficient β (95% CI)*	*P-value*
Model 1	-3.115(-4.723 - -1.508)	< 0.001	-8.876(-11.674 - -6.059)	< 0.001
Model 2	-2.177(-3.756 - − 0.598)	0.007	-7.278(-9.972 - -4.585)	< 0.001
Model 3	-1.712(-3.306 - − 0.118)	0.035	-6.362(-9.055 - -3.670)	< 0.001
**DLCOc N = 2356**	**No COPD (** *N* ** = 2033)**		**COPD (** *N* ** = 323)**	
	*Unstandardized coefficient β (95% CI)*	*P-value*	*Unstandardized coefficient β (95% CI)*	*P-value*
Model 1	− 0.189(-0.308 - − 0.071)	0.002	− 0.675(-1.038 - − 0.312)	<0.001
Model 2	− 0.171(-0.291 - 0.051)	0.005	− 0.643(-1.009 - − 0.278)	<0.001
Model 3	− 0.112(-0.232 - 0.009)	0.070	− 0.550(-0.909 - − 0.191)	0.003
**DLCOc/VA** *N* ** = 2356**	**No COPD** (*N*** = 2033)**		**COPD (** *N* ** = 323)**	
	*Unstandardized coefficient β (95% CI)*	*P-value*	*Unstandardized coefficient β (95% CI)*	*P-value*
Model 1	− 0.002(-0.023 - 0.019)	0.849	− 0.075(-0.128 - − 0.023)	0.005
Model 2	− 0.010(-0.031 - 0.011)	0.343	− 0.074(-0.126 - − 0.022)	0.005
Model 3	0.001(-0.020 - 0.021)	0.939	− 0.065(-0.117 - − 0.014)	0.013

Model 1: Age, sex, Rotterdam Study subcohort adjusted

Model 2: Model 1 + diabetes, physical activity, eGFR, BMI, oral and inhaled corticosteroids prescription adjusted

Model 3: Model 2 + smoking status adjusted

Stratified analyses for SAF with COPD prevalence and lung function by smoking status in total population are shown in Supplemental Tables 4 and 5 respectively. None of the associations between SAF and COPD were significant in 3 smoking subgroups, but betas were largest in never smokers compared to former and current smokers. Regarding lung function, in Model 2, SAF associated with FEV_1_% predicted only in former smokers (β =-4.567[-6.614, -2.521], *p* < 0.001); and with D_LCO_c in former (β =-0.241 [-0.401, -0.081], *p* = 0.003) and current smokers (β=-0.374[-0.678, -0.070], *p* = 0.016 respectively).

Stratified analyses by packyears found SAF was not associated with COPD prevalence in the packyears subgroups (Supplemental Table 6). However, significant inverse associations were observed in 0–10 packyears subgroups with full adjustment for covariates between SAF with FEV_1_% predicted ( β= -6.524 [-10.003, -3.044], *p* < 0.001) and with D_LCO_c( β= -0.393 [-0.645, -0.142], *p* = 0.002) (Model 3, Supplemental Table 7). In contrast, SAF was not associated with D_LCO_c/V_A_ in any subgroup.

### Sensitivity analyses

Sensitivity analysis in non-COPD participants was performed on the association between SAF and lung function stratified by smoking status (Supplemental Table 8). There was a significant inverse association between SAF and FEV_1_% predicted only in former smokers (β=-4.286 [-6.546, -2.025], *p* = 0.007, Model 1; β=-3.095 [-5.326, -0.863], *p* = 0.007, Model 2) and in current smokers in Model 1 with similar effect size as former smokers (β=-4.503 [-8.890, -0.116], *p* = 0.044). No significant associations were found between SAF with D_LCO_c or D_LCO_c/V_A_ in any subgroups except for D_LCO_c in current smokers in Model 1 (β= -0.345 [-0.666,-0.025], *p* = 0.035)

In asthma patients (*N* = 181) excluding COPD patients, no associations between SAF and lung function were found (Supplemental Table 9).

### Discussion

In this large population-based study, SAF was significantly positively associated with COPD prevalence, but significance disappeared after adjusting for smoking status. SAF inversely associated with FEV_1_% predicted and D_LCO_c in the total population with strongest relations within COPD and all current and former smokers. We observed associations of sRAGE and EN-RAGE with prevalent COPD but not with incident COPD. There was no significant association between SAF and asthma prevalence observed in our data.

Our findings are consistent with a previous study where higher SAF was associated with lower FEV_1_/FVC ratios in COPD patients and worse lung function in total population [20] and inversely associated with D_LCO_c and D_LCO_c/VA in their total cohort including healthy controls (*N* = 3889) [45]. They did not study a relation in asthma nor with EN-RAGE and sRAGE or an interaction with smoking.

The inverse association with FEV_1_% predicted was much stronger in COPD than in non-COPD participants, which may be explained by the fact that COPD patients have a more disturbed lung function in relation to SAF and a larger variance in lung function parameters. We also found that SAF associated with COPD severity, suggesting that more COPD-related inflammation may lead to higher AGE accumulation, or that higher AGE burden leads to more disturbed lung function.

We found stronger effects of SAF on lung function in current and former smokers than non-smokers. It was reported that RAGE overexpression in COPD smokers causes increased NF-kB (nuclear factor-KappaB)-dependent inflammation leading to lung function decline [24]. This overexpression of RAGE and cigarette smoke-associated airway inflammation might be irreversible after quitting smoking, contributing to the stronger inverse associations of SAF and lung function.

Previous large scale and genetic association studies linked lower sRAGE to COPD and impaired lung function [46]. In a small case-control study, sRAGE was found to be significantly lower in COPD patients (*N* = 200) [27], consistent with our data. Additionally we found lower serum sRAGE levels associated with higher COPD prevalence which could be explained by its potential protective role as decoy receptor for AGEs and other pro-inflammatory ligands [47, 48]. However, another study with 1454 COPD patients did not find an association between sRAGE and FEV_1_ decline [49]. EN-RAGE has not been extensively studied but previously negatively correlated with FEV_1_% predicted [50]. In our study, higher EN-RAGE was associated with higher COPD prevalence and this could suggest a role in lung inflammation [34]. We also studied sRAGE and EN-RAGE prospectively but found no significant associations with incident COPD. It should be noted that there was a small number of incident cases (*N* = 151). Also sRAGE has the limitation that smoking causes an instant drop in circulating levels [51], which may impact observed associations.

**Fig. 1:**
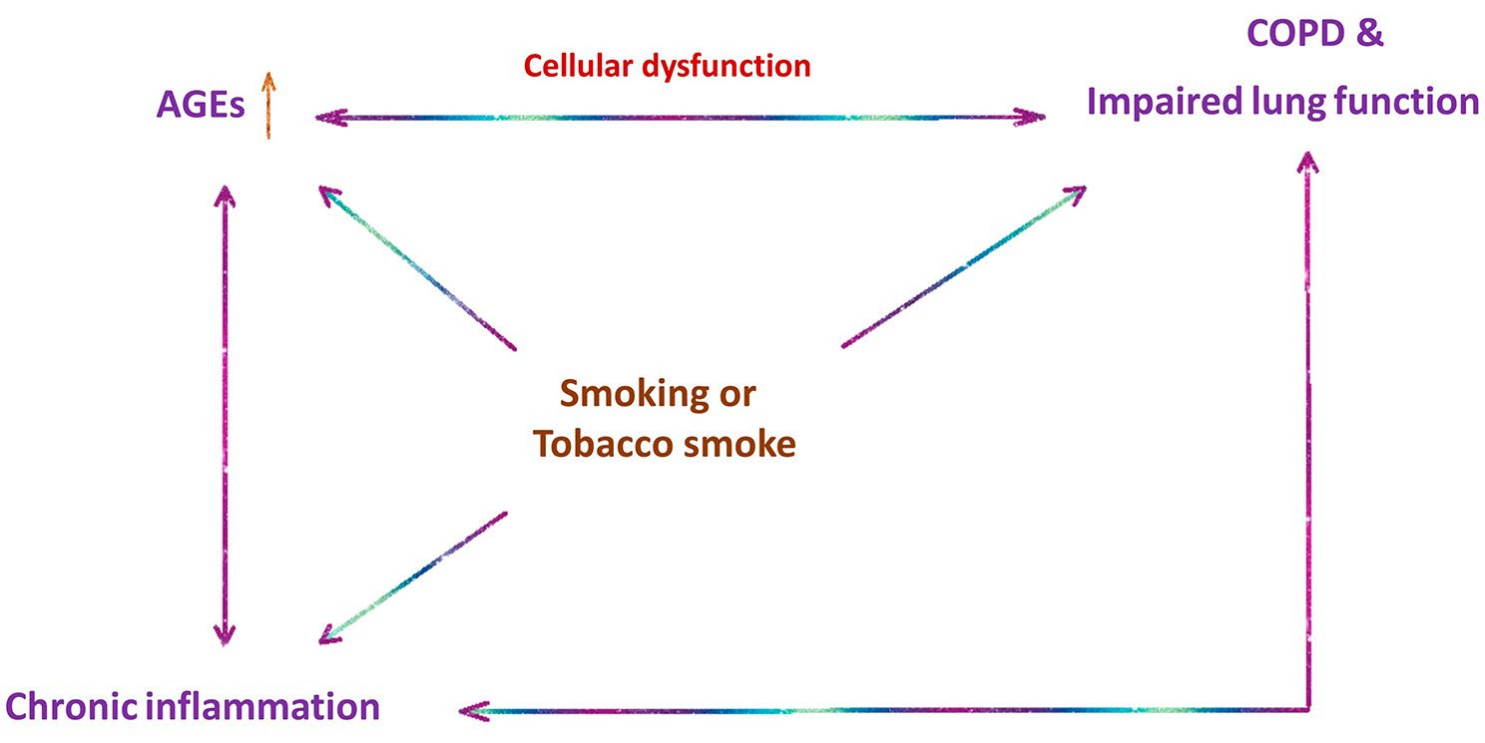
Potential interrelation between AGEs, COPD, and smoking

There are several explanations for our findings: (1) Higher AGEs reflected by SAF impair lung function due to their negative effects on tissues or binding with RAGE; (2) There could be a reversed causation where AGE formation is increased due to the inflammatory status in COPD; (3) Smoking causes both an increase in AGEs and disturbed lung function; (4) A combination of these factors (Fig. 1).

Regarding the first possible explanation that higher AGE accumulation causes airway obstruction, it was found that levels of AGEs and other RAGE ligands such as HMGB1(High mobility group box 1 protein)were higher in lung tissues of COPD patients [52], and RAGE expression was also found significantly higher in healthy human lung tissue compared to 15 other human tissues [53]. We might speculate that increased AGEs could contribute to inflammation in lung pathology by increasing AGE-RAGE axis activity. This way, AGEs could be more harmful for COPD patients by amplifying and activating inflammatory signals. This might also explain that the association between SAF and lung function is stronger within COPD.

Concerning the second explanation of reversed causation: it is also possible that more COPD-associated inflammation increased AGE formation, as was shown for both inflammation and oxidative stress [13, 15], with oxidative stress being the major driving mechanism involved both in COPD [54] and AGE formation.

The third explanation is that associations between SAF and COPD could be explained by the confounding effect of smoking as a major risk factor for COPD and a major source of AGEs accumulation [17]. After adjusting for smoking, the association between SAF and COPD indeed became non-significant. This may also be partly due to the fact that COPD and current and former smoking largely overlapped. However, the associations between SAF and lung functions were still present after adjusting for smoking, although attenuated.

In stratified analyses, the associations were stronger in current and former smokers but not present in non-smokers, even in the non-COPD population. This suggests that smoking is an important effect modifier in the association between SAF and lung function, including persons without compromised lung function. Another explanation could be that smoking is on the causal path where AGEs mediate the link between smoking and lung function. This is supported by reports that on one hand cigarette smoke is a source of toxic reactive glycation products that can rapidly react with proteins to form AGEs [17, 55] and on the other hand AGEs were found higher in lung tissue of COPD patients [52] and SAF was positively associated with COPD severity in our study. Associations of SAF with lung function impairment in former smokers suggest that AGEs accumulation may have long-lasting effects.

We didn’t observe a significant association between SAF and asthma prevalence yet a positive effect size was noticed. The absence of an association could be explained by the small cases number among the total population(n/*N* = 181/1964), or there was no association between the two. Previous studies have focused on the role of RAGE and sRAGE in the pathogenesis of asthma. sRAGE is a proposed emphysema and airflow obstruction biomarker. However, no cohort studies to our knowledge have studied on the association between asthma and AGEs, as a main ligand of the AGE-RAGE axis.

Several limitations of our study need to be noted. The RS is a population-based cohort of middle-aged and elderly subjects. Participants continuing to come to the research center could potentially be “healthier” than those who did not. Another limitation is that no follow-up SAF measurements are available to investigate longitudinal relationships between COPD and SAF. We were also not able to exclude a potential direct effect of cigarette smoke on the skin of the dominant arm and SAF value. Besides, if SAF reflects the status of different types of AGEs in lung tissue remains unclear. Other limitations are small sample sizes for analyses in subgroups and asthma but this is the first study investing such relations. We cannot exclude the possibility of residual confounding and selective survival. Due to the cross-sectional nature of our study we cannot conclude on causality or direction of observed associations.

The strengths are that we used a well-phenotyped population-based cohort, with a large sample size of COPD participants and the possibility to study the role of smoking in the relationship between AGEs and impairment of lung functions both in the total population and in (non)COPD subgroups.

In conclusion, skin AGEs are associated with COPD prevalence, COPD severity evaluated by GOLD stage and impairment of lung function. Our findings suggest that smoking plays an important role in these associations but its exact role has to be investigated in further studies. Future large scale prospective studies and Mendelian randomization studies may aid in deciphering the causal chain between smoking, AGEs, lung function, and COPD.

### Electronic supplementary material

Below is the link to the electronic supplementary material.

Supplementary Material 1

## Data Availability

The datasets generated and/or analysed during the current study are not publicly available due to restrictions based on privacy regulations and informed consent of the participants but are available from the management team of the Rotterdam Study (secretariat.epi@erasmusmc.nl) on reasonable request.
