# Supplementary material for: The AGE-RAGE axis associates with chronic pulmonary diseases and smoking in the Rotterdam study
Source: Respir Res. 2024 Feb 9;25:85. doi: 10.1186/s12931-024-02698-1 (PMC10858545; doi:10.1186/s12931-024-02698-1)
Supplement: Supplementary file 1 — Supplementary Material 1 [file 12931_2024_2698_MOESM1_ESM.docx]

**Supplemental Tables**

**Supplementary Table 1** Characteristics of the study population Stratified by Rotterdam Study Cohort

| Parameters | Total population | RS I | RS II | RS III |
| --- | --- | --- | --- | --- |
| N | 2577 | 615 (23.9%) | 923 (35.8%) | 1039 (40.3%) |
| Age (years)* | 72.28 ± 9.29 | 82.54 ± 3.90 | 75.63 ± 3.75 | 63.24 ± 6.20 |
| Male/n (%) | 1148 (44.5%) | 260 (42.3%) | 428 (46.4%) | 460 (44.3%) |
| Female/n (%) | 1429 (55.5%) | 355 (57.7%) | 495 (53.6%) | 579 (55.7%) |
| SAF* | 2.39 ± 0.49 | 2.50 ± 0.51 | 2.47 ± 0.50 | 2.26 ± 0.43 |
| COPD | 613 (23.8%) | 213 (34.6%) | 246 (26.7%) | 154 (14.8%) |
| Asthma | 215 (8.3%) | 51 (8.3%) | 94 (10.2%) | 70 (6.7%) |
| Diabetes （N=389） | 389 (15.1%) | 103 (16.7%) | 175 (19.0%) | 111 (10.7%) |

* Among RS I, RS II and RS III, p-value < 0.001 obtained from Anova

**Supplementary Table 2** Characteristics of the study population stratified by smoking status

| Parameters | Total population | Never Smokers | Ex-smokers | Current Smokers |
| --- | --- | --- | --- | --- |
| N | 2575 | 848 (32.9%) | 1483 (57.6%) | 244 (9.5%) |
| Age(years)* | 72.28 ± 9.29 | 71.86 ± 9.40 | 73.35 ± 9.01 | 67.22 ± 8.78 |
| Sex(male,%) | 1146 | 265 (31.3%) | 736 (52.1%) | 183 (44.7%) |
| SAF* | 2.39 ± 0.49 | 2.30 ± 0.46 | 2.42 ± 0.48 | 2.53 ± 0.57 |
| COPD (% in total) | 613 (23.8%) | 106 (12.5%) | 409 (27.6%) | 98 (40.2%) |
| Asthma (% in total) | 215 (8.3%) | 73 (8.6%) | 128 (8.6%) | 14 (5.7%) |
| T2DM (% in total)  (N=2576) | 387 (15.0%) | 117 (13.8%) | 246 (16.6%) | 24 (9.8%) |
| FEV1% predicted* | 97.89 ± 18.36 | 101.63 ± 16.51 | 97.06 ± 18.83 | 89.79 ± 18.53 |
| DLCOc | 7.82 ± 1.71 | 7.91 ± 1.75 | 7.82 ± 1.70 | 7.52 ± 1.64 |
| DLCOc/V_A_* | 1.51 ± 0.23 | 1.56 ± 0.20 | 1.49 ± 0.24 | 1.42 ± 0.24 |

* Among Never smokers, Ex-smokers and Current smokers, p-value < 0.001 obtained from Anova

**Supplementary Table 3** Characteristics of the study population stratified by GOLD Stage

| Parameters | Total population | GOLD stage 1 | GOLD stage 2 | GOLD stage 3&4 |
| --- | --- | --- | --- | --- |
| N | 613 | 390 (63.6%) | 197 (32.1%) | 26 (4.2%) |
| Age(years) | 75.29 ± 8.72 | 75.10 ± 8.61 | 75.79 ± 8.93 | 74.37 ± 9.02 |
| SAF* | 2.50 ± 0.52 | 2.41 ± 0.49 | 2.64 ± 0.53 | 2.78 ± 0.54 |
| FEV1% predicted* | 83.91 ± 18.25 | 94.88 ± 10.37 | 68.07 ± 8.12 | 39.40 ± 6.72 |
| DLCOc* | 7.63 ± 1.83  N=515 | 7.97 ± 1.78  N=340 | 7.06 ± 1.62  N=160 | 5.94 ± 2.58  N=15 |
| DLCOc/VA* | 1.40 ± 0.24  N=515 | 1.40 ± 0.22  N=340 | 1.41 ± 0.27  N=160 | 1.15 ± 0.34  N=15 |

*

Among GOLD stage 1, GOLD stage 2, GOLD stage 3&4, p-value < 0.001 obtained from Anova and Chi-Square

***Stratified analyses***

**Supplementary Table 4** Logistic regression of the association between SAF and COPD stratified by smoking

status (N=2575)

| **Never Smokers**  **COPD (n/N=106/ 848)** | | | **Ex-smokers**  **COPD (n/N=409/1483)** | | **Current Smokers COPD (n/N=98/244)** |  |  |
| --- | --- | --- | --- | --- | --- | --- | --- |
|  | **Exp(B) (95% CI)** | **P-value** | **Exp(B) (95% CI)** | **P-value** | **Exp(B) (95% CI)** | **P-value** |  |
| Model 1 | 1.243(.781 – 1.980) | .359 | 1.047(.810 - 1.353) | .726 | 1.146(.702 - 1.870) | .586 |  |
| Model 2 | 1.410(.876 - 2.272) | .157 | 0.998(.765 - 1.302) | .989 | 1.131(.667 - 1.917) | .647 |  |

Model 1: Age, sex, Rotterdam Study subcohort adjusted

Model 2: Model 1 + diabetes, physical activity, eGFR, BMI, oral and inhaled corticosteroids prescription adjusted

**Supplementary Table 5** Linear regression of the association between SAF and lung function parameters

stratified by smoking status in total population

| **FEV_1_%predicted** **N=2575** | | **NeverSmokers (N=848)**  **(COPD patients=106)** | | **Ex-smokers (N=1483)**  **(COPD patients=409)** | | **Current smokers (N=244)**  **(COPD patients=98)** | |
| --- | --- | --- | --- | --- | --- | --- | --- |
|  | | *Unstandardized coefficient β (95% CI)* | *P-value* | *Unstandardized coefficient β (95% CI)* | *P-value* | *Unstandardized coefficient β (95% CI)* | *P-value* |
| Model 1 | -.929(-3.540 - 1.682) | .485 | -6.394(-8.498 - -4.290) | < 0.001 | -4.674(-8.989 - -.358) | .034 |  |
| Model 2 | -.493(-3.057 - 2.071) | .706 | -4.567(-6.614 - -2.521) | < 0.001 | -3.798(-7.961 - .365) | .074 |  |
| **D_LCO_c**  **N=2434** | **Never Smokers (N=790)**  **(COPD patients=87)** | | **Ex-smokers (N=1403)**  **(COPD patients=341)** | | **Current smokers (N=241)**  **(COPD patients=86)** | |  |
|  | *Unstandardized coefficient β (95% CI)* | *P-value* | *Unstandardized coefficient β (95% CI)* | *P-value* | *Unstandardized coefficient β (95% CI)* | *P-value* |  |
| Model 1 | -.070(-.258 - .118) | .467 | -.295(-.453 - -.138) | < 0.001 | -.411(-.717 - -.105) | .009 |  |
| Model 2 | -.056(-.247 - .136) | .569 | -.241(-.401 - -.081) | 0.003 | -.374(-.678 - -.070) | .016 |  |
| **D_LCO_c/V_A_ N=2434** | **Never Smokers (N=790)**  **(COPD patients=87)** | | **Ex-smokers (N=1403)**  **(COPD patients=341)** | | **Current smokers (N=241)**  **(COPD patients=86)** | |  |
|  | *Unstandardized coefficient β (95% CI)* | *P-value* | *Unstandardized coefficient β (95% CI)* | *P-value* | *Unstandardized coefficient β (95% CI)* | *P-value* |  |
| Model 1 | .019(-.014 - .052) | .267 | -.018(-.045 - .009) | 0.196 | -.047(-.101 - .007) | .087 |  |
| Model 2 | .014(-.018 - .047) | .389 | -.023(-.049 - .004) | 0.096 | -.048(-.099 - .003) | .065 |  |

Model 1: Age, sex, Rotterdam Study subcohort adjusted

Model 2: Model 1 + diabetes, physical activity, eGFR, BMI, oral and inhaled corticosteroids prescription adjusted

**Supplementary Table 6** Logistic regression of the association between SAF and COPD stratified by

packyears in total population

| **COPD (n/N=595/2532)** | **Model 1** | | **Model 2** | | **Model 3** | |
| --- | --- | --- | --- | --- | --- | --- |
|  | *Exp(B) (95% CI)* | *P-value* | *Exp(B) (95% CI)* | *P-value* | *Exp(B) (95% CI)* | *P-value* |
| Never smokers  (N=850)  COPD N=107 | 1.261 (.793 - 2.004) | .327 | 1.429 (.889 - 2.298) | .141 | 1.416 (.879 - 2.280) | .153 |
| 0-10 packyears  (N=587)  COPD N=119 | 1.118 (.683 - 1.830) | .658 | 1.139 (.679 - 1.910) | .621 | 1.090 (.649 - 1.831) | .746 |
| 10-30 packyears  (N=577)  COPD N=153 | 1.041 (.696 - 1.555) | .846 | .992 (.654 - 1.504) | .968 | .809 (.524 - 1.247) | .337 |
| >30 packyears  (N=518)  COPD N=216 | 1.129 (.800 - 1.594) | .490 | 1.096 (.767 - 1.566) | .613 | 1.052 (.733 - 1.509) | .784 |

Model 1: Age, sex, Rotterdam Study subcohort adjusted

Model 2: Model 1 + diabetes, physical activity, eGFR, BMI, oral and inhaled corticosteroids prescription adjusted

Model 3: Model 2 + smoking status adjusted

|  | **Model 1** | | **Model 2** | | **Model 3** | |
| --- | --- | --- | --- | --- | --- | --- |
| **FEV_1_ %predicted**  **(N=2532)** | *Unstandardized coefficient β (95% CI)* | *P-value* | *Unstandardized coefficient β (95% CI)* | *P-value* | *Unstandardized coefficient β (95% CI)* | *P-value* |
| Never smokers  (N=850) | -1.012 (-3.625 - 1.601) | .447 | -.595 (-3.163 – 1.973) | .650 | -.512 (-3.079 – 2.056) | .696 |
| 0-10 packyears  (N=587) | -7.680 (-11.145 - -4.215) | <.001 | -6.832 (-10.288 - -3.376) | <.001 | -6.524 (-10.003 - -3.044) | <.001 |
| 10-30 packyears  (N=577) | -5.065 (-8.293 - -1.837) | .002 | -4.097 (-7.260 - -.934) | .011 | -2.999 (-6.177 - .179) | .064 |
| >30 packyears  (N=518) | -4.603 (- 7.777 - - 1.428) | .005 | -2.997 (-6.052 - .058) | .055 | -2.399 (-5.468 - .671) | .125 |
| **D_Lco_c (N=2400)** | *Unstandardized coefficient β (95% CI)* | *P-value* | *Unstandardized coefficient β (95% CI)* | *P-value* | *Unstandardized coefficient β (95% CI)* | *P-value* |
| Never smokers  (N=793) | -.069 (-.257 - .119) | .471 | -.055 (-.246 - .136) | .571 | -.057 (-.248 - .135) | .562 |
| 0-10 packyears  (N=558) | -.415 (-.659 - -.170) | <.001 | -.402 (-.651 - -.153) | .002 | -.393 (-.645 - -.142) | .002 |
| 10-30 packyears  (N=553) | -.363 (-.601 - -.124) | .003 | -.251 (-.491 - -.010) | .041 | -.177 (-.419 - .066) | .153 |
| >30 packyears  (N=496) | -.176 (-.426 - .073) | .165 | -.165 (-.417 - .087) | .199 | -.118 (-.372 - .137) | .365 |
| **D_LCO_c/V_A_ (N=2400)** | *Unstandardized coefficient β (95% CI)* | *P-value* | *Unstandardized coefficient β (95% CI)* | *P-value* | *Unstandardized coefficient β (95% CI)* | *P-value* |
| Never smokers  (N=793) | .020 (-.014 - .053) | .245 | .016 (-.017 - .048) | .354 | .015 (-.018 - .048) | .363 |
| 0-10 packyears  (N=558) | -.007 (-.051 - .036) | .734 | -.018 (-.060 - .024) | .393 | -.012 (-.054 - .030) | .577 |
| 10-30 packyears  (N=553) | -.053 (-.096 - -.011) | .013 | -.035 (-.076 - .005) | .087 | -.021 (-.062 - .020) | .308 |
| >30 packyears  (N=496) | -.028 (-.071 - .016) | .210 | -.036 (-.078 - .006) | .091 | -.032 (-.074 - .011) | .144 |

**Supplementary Table 7** Linear regression of the association between SAF and lung function parameters

stratified by packyears in total population

Model 1: Age, sex, Rotterdam Study subcohort adjusted

Model 2: Model 1 + diabetes, physical activity, eGFR, BMI, oral and inhaled corticosteroids prescription adjusted

Model 3: Model 2 + smoking status adjusted

***Sensitivity analyses***

**Supplementary Table 8** Linear regression of the association between SAF and lung function parameters

stratified by smoking status in non-COPD participants

| **FEV1 predicted%** **N=1962** | **Never Smokers (N=742)** | | **Ex-smokers (N=1074)** | | **Current smokers (N=146)** | |
| --- | --- | --- | --- | --- | --- | --- |
|  | *Unstandardized coefficient β (95% CI)* | *P-value* | *Unstandardized coefficient β (95% CI)* | *P-value* | *Unstandardized coefficient β (95% CI)* | *P-value* |
| Model 1 | .220 (-2.509 – 2.950) | .874 | -4.286 (-6.546 - -2.025) | < 0.001 | -4.503 (-8.890 - -.116) | .044 |
| Model 2 | .909 (-1.749 – 3.567) | .502 | -3.095 (-5.326 - -.863) | .007 | -3.461 (-7.935 – 1.013) | .128 |
| **D_LCO_c N=2030** | **Never Smokers (N=716)** | | **Ex-smokers (N=1147)** | | **Current smokers (N=167)** | |
|  | *Unstandardized coefficient β (95% CI)* | *P-value* | *Unstandardized coefficient β (95% CI)* | *P-value* | *Unstandardized coefficient β (95% CI)* | *P-value* |
| Model 1 | -.040 (-.240 - .161) | .698 | -.128 (-.292 - .037) | .129 | -.345 (-.666 - -.025) | .035 |
| Model 2 | -.021 (-.224 - .182) | .839 | -.099 (-.268 - .069) | .248 | -.304 (-.628 - .019) | .065 |
| **D_LCO_c/VA N=2030** | **Never Smokers (N=716)** | | **Ex-smokers (1147)** | | **Current smokers (N=167)** | |
|  | *Unstandardized coefficient β (95% CI)* | *P-value* | *Unstandardized coefficient β (95% CI)* | *P-value* | *Unstandardized coefficient β (95% CI)* | *P-value* |
| Model 1 | .020 (-.016 - .057) | .274 | .012 (-.017 - .042) | .402 | -.032 (-.091 - .028) | .295 |
| Model 2 | .014 (-.022 - .050) | .438 | .001 (-.027 - .030) | .928 | -.032 (-.090 - .025) | .266 |

Model 1: Age, sex, Rotterdam Study subcohort adjusted

Model 2: Model 1 + diabetes, physical activity, eGFR, BMI, oral and inhaled corticosteroids prescription adjusted

**Supplementary Table 9** Linear regression of the association between SAF and lung function parameters in

asthma patients (COPD patients excluded)

|  | **Asthma patients(N=181)** | |
| --- | --- | --- |
| **FEV1%predicted** | *Unstandardized coefficient β (95% CI)* | *P-value* |
| Model 1 | -3.741 (-9.443 - 1.961) | .197 |
| Model 2 | -2.132 (-7.828 - 3.563) | .461 |
| Model 3 | -2.362 (-8.121 - 3.397) | .419 |
|  | **Asthma patients (N=159)** | |
| **D_LCO_c** | *Unstandardized coefficient β (95% CI)* | *P-value* |
| Model 1 | .012 (-.433 - .457) | .957 |
| Model 2 | -.038 (-.508 - .432) | .874 |
| Model 3 | .028 (-.451 - .507) | .907 |
|  | **Asthma patients (N=159)** | |
| **D_LCO_c/V_A_** | *Unstandardized coefficient β (95% CI)* | *P-value* |
| Model 1 | -.029 (-.103 - .044) | .431 |
| Model 2 | -.041 (-.114 - .033) | .275 |
| Model 3 | -.028 (-.103 - .046) | .452 |

Model 1: Age, sex, Rotterdam Study subcohort adjusted

Model 2: Model 1 + diabetes, physical activity, eGFR, BMI, oral and inhaled corticosteroids prescription adjusted

Model 3: Model 2 + smoking status adjusted

**
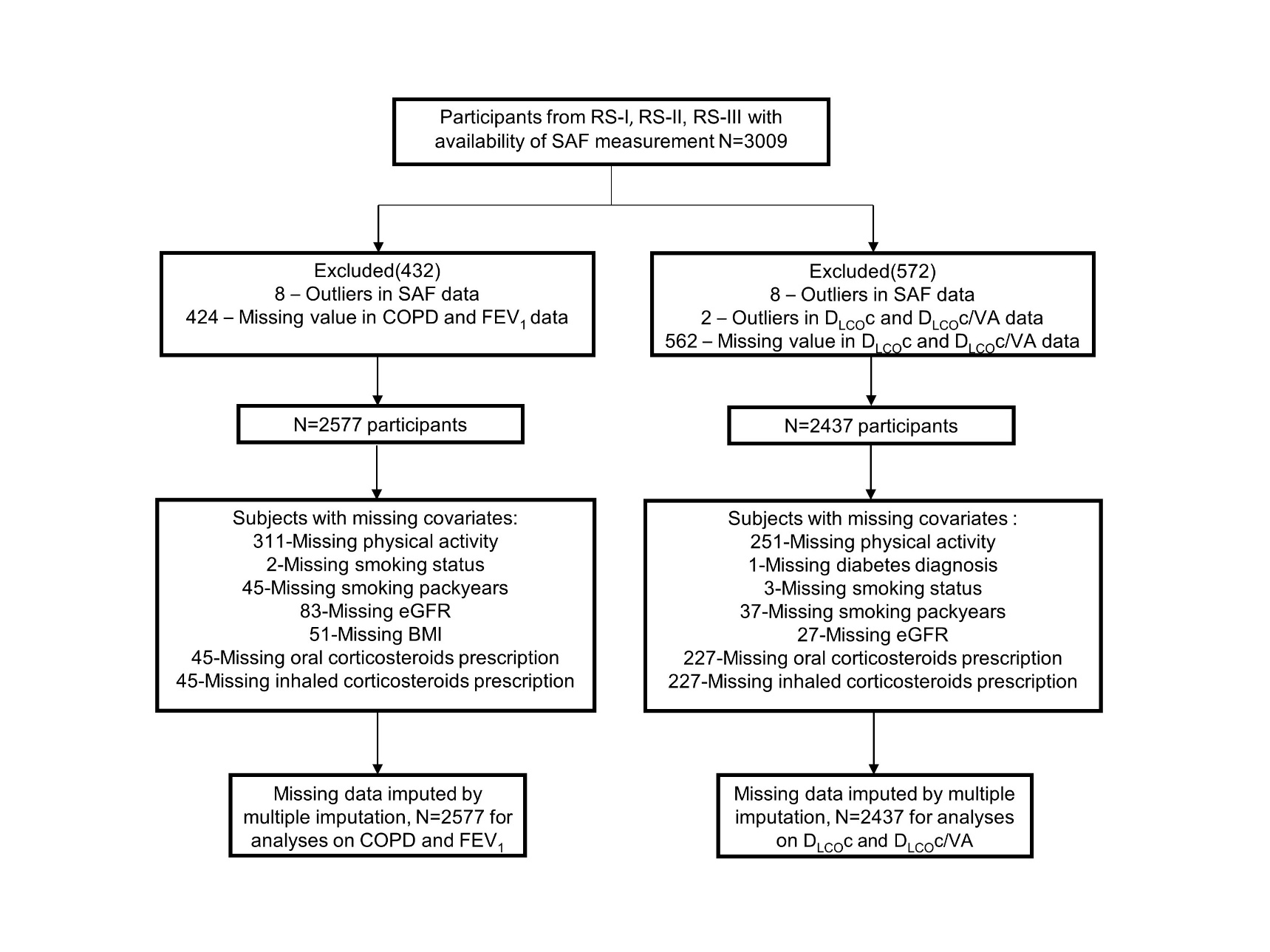
Supplementary Figure 1**. Flowchart of data preparation for linear and logistic regressions


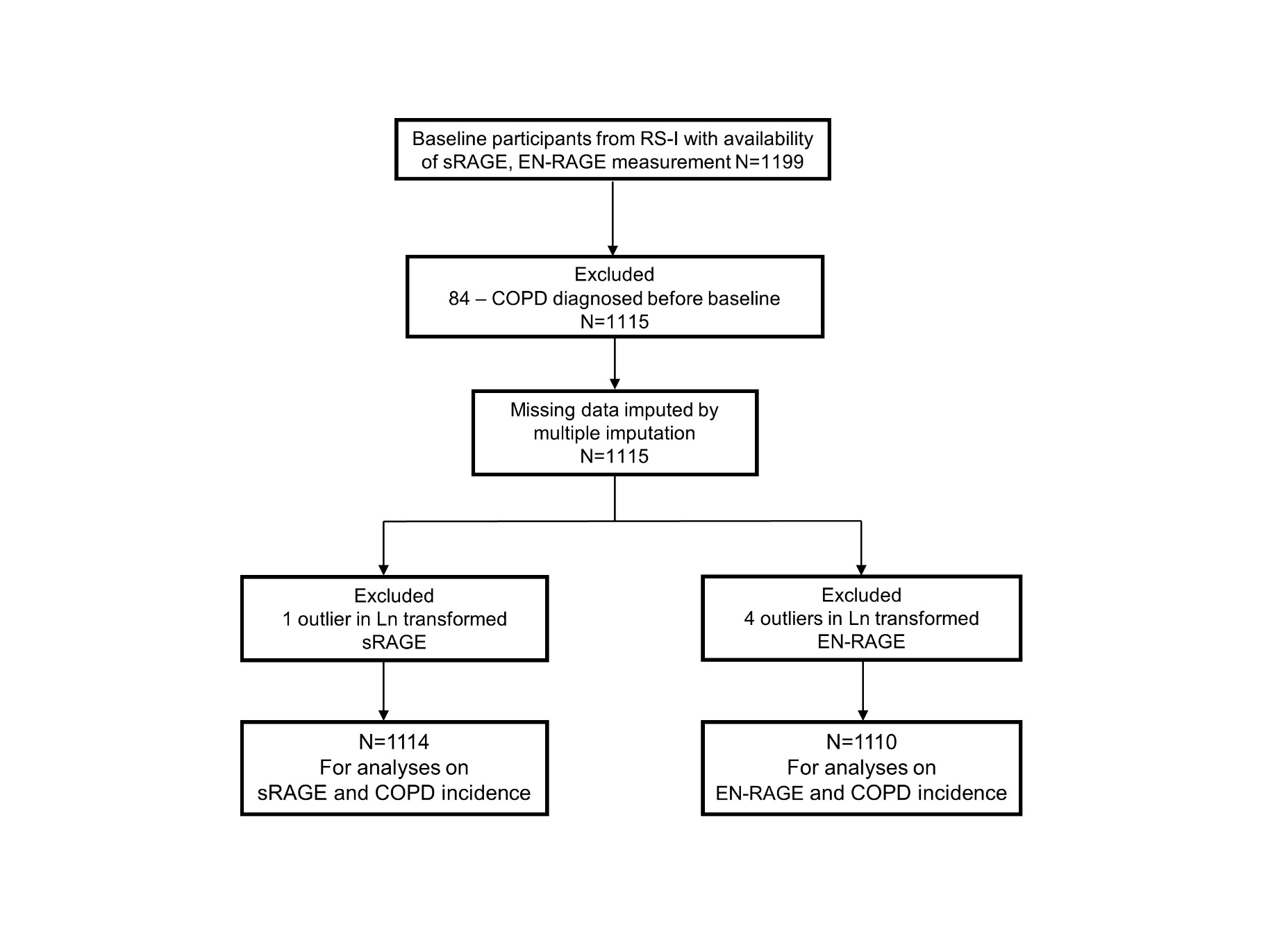
**Supplementary Figure 2.** Flowchart of data preparation for Cox proportional analyses
